# Future directions for reproductive coercion and abuse research

**DOI:** 10.1186/s12978-022-01550-3

**Published:** 2023-01-02

**Authors:** Karen Trister Grace, Elizabeth Miller

**Affiliations:** 1 School of Nursing, College of Public Health, George Mason University, 4400 University Drive, Mailstop 3C4, Fairfax, VA 22030 USA; 2 Adolescent and Young Adult Medicine, University of Pittsburgh, 120 Lytton Avenue, Pittsburgh, PA 15213-1481 USA

**Keywords:** Reproductive coercion and abuse, Intimate partner violence, Sexual violence

## Abstract

**Background:**

Reproductive coercion and abuse (RCA) is a form of intimate partner violence (IPV) in which people with the capacity for pregnancy experience coercive behaviors that threaten their reproductive autonomy. Behaviors that constitute RCA include contraceptive control/sabotage, pregnancy pressure, and controlling the outcome of a pregnancy.

**Summary:**

Several areas of RCA study have emerged: associations with IPV, health outcomes resulting from RCA, and demographic and contextual factors associated with experiencing RCA. Current research in these areas is summarized and placed in a global context, including sexual and gender minority groups, use of RCA (exploring perpetration), RCA interventions, RCA in women with disabilities, and the question of whether people assigned male at birth can be RCA victims.

**Conclusion:**

Areas for future exploration include evolving interpretations of pregnancy intention in the setting of fewer options for abortion, RCA in people with disabilities and multiple levels of marginalization, including sexual and gender minorities; intersections between RCA and economic abuse in the context of efforts at economic justice; and community-centered approaches to intervention and prevention.

## Background

Reproductive capacity is legislated and controlled by governmental systems, healthcare providers, and individuals on a global scale and throughout history. The treatment, representation, and control of particular bodies (gendered, racialized, impoverished, disabled) reflects the legacy of white supremacy and misogyny that impacts all levels of society. On an interpersonal level, use of coercive behaviors to control reproductive health decisions is perpetrated by intimate partners, family members and in-laws [1–3] as well as healthcare providers [4–6]. This commentary will limit its focus to the perspectives of people who have experienced coercion of reproductive health decisions from an intimate partner, referred to as reproductive coercion (RC), a form of intimate partner violence (IPV) in which people with the capacity for pregnancy experience coercive behaviors that threaten their reproductive autonomy. Behaviors that constitute RC include contraceptive sabotage, pregnancy pressure, and controlling the outcome of a pregnancy. Terminology in the field of RC research has also evolved to a more widely used term “reproductive coercion and abuse” (RCA), in an attempt to clarify the boundaries to be limited to interpersonal behaviors, and not coercive state policies at the structural or systemic level, such as forced sterilization or restricting access to abortion. This coercion is certainly harmful, and is a different phenomenon from RC/RCA, and different interventions are indicated. The remainder of this commentary will use the term reproductive coercion and abuse (RCA).

Although RCA behaviors were evident in the IPV literature prior to 2010 [7–11], focused research on RCA specifically highlighted a distinct set of behaviors that were associated with poor sexual and reproductive health outcomes. These include pregnancies that are mistimed, unplanned, and undesired (“unintended pregnancy”), regardless of whether the RCA occurred in the context of other forms of IPV such as physical and sexual violence. Research over the past decade has included refinement of this construct [12, 13], evaluations of assessment and interventions for RCA [14, 15] as well as epidemiologic and descriptive research to elucidate risks, outcomes, and associated factors [16–19]. Several areas of RCA study have emerged: associations with IPV, health outcomes resulting from RCA, and demographic and contextual factors associated with experiencing RCA. A brief summary of these areas of RCA research is provided, and we also summarize emerging areas of research to inform future work in this area. Emerging areas include RCA in sexual and gender minority groups, exploring RCA perpetration, and RCA interventions.

## Associations with intimate partner violence and sexual violence

Whether RCA is a separate but overlapping conceptual entity from IPV or one vehicle in the broader constellation of IPV tactics has been debated by researchers [13]. IPV and RCA are closely associated in epidemiologic studies. RCA is associated with homicide risk [20] and other severe forms of violence such as sexual assault, stalking, traumatic brain injury, and polyvictimization [13, 16, 20–22]. Tactics of IPV perpetrators vary widely, including physical assault, threatening to have an undocumented partner deported, threatening pets, rape, withholding money, cyberstalking, using religious teachings or traditions as means of control [20], and a multitude of other behaviors; the central motivating factors for such behaviors are a desire for power and control [23]. RCA similarly includes a range of controlling behaviors including violence and threats of violence if the survivor uses contraception, monitoring menstrual cycles, and refusing to provide the money to purchase contraception [24]. A distinguishing factor between IPV and RCA is that while RCA may include a variety of violent and coercive tactics, purely IPV behaviors do not include a goal of reproductive control.

In studies with samples of IPV survivors, some risk factors emerge for experiencing RCA, including having smaller family size and not having children with the abusive partner [25]. This may be a result of a partner seeking to solidify an unstable relationship, or it may be that people who already have children with their abusive partners are simply less vulnerable to RCA, because this means of gaining power and control over a partner has already been established. Studies have also demonstrated that RCA may be experienced even without being exposed to physical or sexual IPV [17].

The association of RCA with other forms of IPV also extends to sexual violence in non-partner sexual relationships, specifically when sperm-producing sexual partners refuse to use a condom, remove a condom during sex (called ‘stealthing’), or manipulate the condom by poking holes or tearing the condom [26]. As RCA items have been validated and refined [12], behaviors related to condom nonuse or manipulation appear to be a particularly salient set of behaviors that are critical for understanding not only pregnancies that are undesired, but also increased risk for HIV and other STI transmission.

## Associations with demographic characteristics

Approximately 8% of woman-identified respondents report RCA in American population-based data [27], and lifetime prevalence is as high as 25–37% in community and clinic-based samples [28, 29]. A number of demographic and contextual characteristics have emerged in the literature as frequently correlated with the experience of RCA. In the US, these include having higher number of current or lifetime sex partners [30–35], being single [36–38], and races other than White [16, 20, 27, 28, 32, 36, 38–40]. In the global health literature, risk is associated with smaller family size [2, 3], having no formal education [41], poverty [42], and partners who have other concurrent partners [43]. Some of these identified sociodemographic characteristics associated with RCA need further explication to elucidate the underlying mechanisms by which exposure to poverty or having a minoritized background may contribute to elevated risk for RCA. Additionally, such characteristics may have potential to inform clinical suspicion for people who may benefit from additional assessment or the availability of less detectable methods of contraception, although caution is needed to reduce implicit biases among providers. Many questions remain about who may be made more vulnerable to exposure to RCA and how best to mitigate harm from these behaviors, including elucidating more survivor-centered harm reduction strategies and options for optimizing their health and well-being.

## Health outcomes

A number of health outcomes are associated with experiencing RCA, making this a critical area to address for improvement in public health. Health consequences include STIs [35, 44], mental health symptoms (depression, anxiety and PTSD) [29–31, 45], negative birth outcomes such as low birthweight [46], and unintended pregnancy [17, 37, 39], which is associated with additional negative health outcomes for parents and children [47–52]. Behaviors with potential to increase risk for poor health outcomes are also associated with RCA, such as having multiple sexual partners [18, 30, 31, 33–35, 53, 54], early sexual initiation [31, 32], and decreased condom negotiation skills [30, 55]. Substance use is also correlated with experiences of RCA; people who experienced RCA were more likely to report past month smoking or drug use [32], and had higher odds of alcohol or drug use prior to sex [22]. In studies conducted in clinical settings, RCA exposure is associated with seeking care for reproductive and sexual health concerns including frequent use of emergency contraception and requests for pregnancy and STI testing, indicating opportunity for assessment and intervention [32, 56].

## Summary of RCA research to date in select key areas and implications for future research

### Sexual and gender minority groups

The elevated risk for exposure to violence among people who identify as sexual and gender minority is well documented [57–59], and associated with marginalization, stigma, and persistent experiences of discrimination (including homophobia, transphobia, misogyny, ableism, and racism). RCA is another form of violence that appears to be more prevalent among these groups, though findings have been conflicting. Many studies include sexual and gender minority participants, but do not report findings stratified by these groups [60–64]. Most studies that focus on sexual and gender minority individuals examine behaviorally bisexual women, or women who have sex with women and men (WSWM), who also are found in many studies to experience additional sexual and reproductive health risks, such as STIs, unwanted pregnancies, unprotected sex, and health risks associated with the marginalization and criminalization of transactional sex [65]. Several studies found that WSWM were significantly more likely to experience RCA from a male partner than women who exclusively have sex with men (WSM) [32, 66, 67], even controlling for IPV [65]. Non-significant findings relating to RCA among sexual and gender minority participants have also been reported, including studies of sexually active adolescents [68], youth in foster care [22], college students [21, 69], and family planning clinic patients [70]. To our knowledge, only one qualitative study explores RCA among sexual and gender minority participants specifically. A study of Black WSWM explored control and coercion of reproductive decision-making of women with feminine appearance and roles by female partners with more masculine presentations of self, suggesting this coercive behavior contributed to the latter’s gender identity and community authority [71].

### Use of RCA—exploring perpetration

Few studies have examined prevalence or correlates of RCA perpetration. In the US, in a sample of 39 predominantly non-Hispanic Black men in health clinics, 12.8% ever engaged in one or more RCA behaviors, and the most common behavior reported was failing to withdraw when withdrawal was the agreed-upon contraceptive method [72]. In a sample of 477 college students who were assigned male sex at birth, 2.3% reported using RCA in the past 4 months and perpetrators reported significantly more lifetime sexual partners, were less likely to use condoms, and more likely to report using sexual violence against others [32]. Finally, 8% of men in an intervention program for those who have used IPV also reported using RCA to get a partner pregnant [73]. Global research in this area includes one study that included 130 male participants in Papua New Guinea, in which 24.6% of men stated the use of violence was justified if a woman was unable to achieve pregnancy [74]. Beyond dyadic relationships, studies have also identified the significant impact of in-laws in perpetrating RCA. In one study of 717 couple/mothers-in-law triads in Pakistan, 21% of mothers-in-law reported forbidding daughters-in-law from using contraception [75].

Qualitative studies on how and why individuals may use RCA are similarly limited. In one study of 25 young adult Black men, participants discussed RCA as a form of dominance over female partners as well as a sign of attraction [76]. In a qualitative study of 58 low income men, participants discussed seeking connection and permanence in relationships as reasons for engaging in RCA behaviors [77]. A qualitative narrative analysis of one White man’s RCA experience described desire for legacy related to hypermasculinity [78]. And some global studies examine motivations of mother-in-law perpetrators, including a study of mothers-in-law in India in which participants expressed a belief that fertility and sterilization decisions should be made exclusively by the mother-in-law [1]. When people who experience RCA have been asked for their perspectives on what may have motivated the behaviors, they suggest seeking permanent connection due to housing instability and impending incarceration [79–81].

### Interventions to address RCA

Healthcare providers and IPV advocates are challenged with implementing evidence-based interventions to effectively prevent and respond to RCA and promote safety. Recommendations to assess for RCA at periodic intervals and create an environment conducive to disclosure are similar to those developed for IPV. Recent interventions shift away from screening and disclosure-driven practices to more survivor-centered approaches that offer universal education about IPV and RCA regardless of disclosure, including education about harm reduction strategies such as offering methods of contraception that a partner may be less likely to detect and offering referrals to victim service advocates [82, 83]. Whether these strategies to reduce harm ultimately promote or are a detriment to safety has not been evaluated in longitudinal studies. Several studies have evaluated clinic-level interventions intended to enhance or improve RCA assessment, so that providers are more comfortable discussing IPV and RCA and connecting patients who disclose to relevant supports and services. Implementation of psychoeducational scripts (Trauma-Informed Personalized Scripts (TIPS)) tailored for use by providers in family planning clinics did increase discussion about IPV and RCA during the visit, although did not prompt more RCA disclosure [84]. A nurse-delivered intervention in Mexico City, consisting of enhanced IPV screening, referrals, safety planning and follow-up, showed minimal impact on IPV but did significantly reduce incidence of RCA [85]. In studies that emphasize screening for IPV and RCA in clinical practices, recommendations include ensuring that screening occurs in private (away from partners) [86], at multiple visits [87], and offering enhanced training in and organizational support for RCA screening [88–91].

A larger body of research evaluates the Addressing Reproductive Coercion in Health Settings (ARCHES) intervention, which consists of enhanced training of healthcare providers for RCA universal education and brief counseling. A pilot study of ARCHES with family planning counselors found the intervention reduced pregnancy coercion (an aspect of RCA) and increased likelihood of ending an unsafe relationship [14]. A full-scale study with over 4000 participants across 25 family planning clinics did not show a significant impact on RCA in intent-totreat analyses; women experiencing multiple forms of RCA at baseline reported significantly less RCA one year later [92]. A study of an IPV/RCA training program for providers focused on communication skills for sensitive topics using simulation and role-playing as compared to the training in the ARCHES program, found all types of training improved provider communication, but no increased benefit to the enhanced training [93]. Qualitative data on ARCHES implementation supports the usefulness of the intervention in increasing confidence to offer universal education and brief counseling related to IPV/RCA, and also supports acceptability of the intervention to patients, while identifying systemic barriers to implementation [15]. Project Connect, a multi-state public health demonstration project for the implementation of ARCHES, providing RCA training and screening tools for providers, has also been evaluated. A pilot study with 47 providers to assess quality of implementation confirmed utility and acceptability of the intervention [89]. Implementation was also evaluated with family planning clinic patients and providers, supporting acceptability and value of the intervention as well as increases in provider knowledge [94].

One IPV intervention has been studied in terms of its effectiveness in reducing RCA, offering a promising solution for future development. The technology-based IPV intervention, called myPlan, was also shown to significantly reduce RCA in a sample of college students, though the intervention was not specifically tailored to RCA [95].

### RCA in women with disabilities

Very few studies to date have explored how RCA may be experienced among women with disabilities. One qualitative study examined IPV survivors with disabilities who experienced unintended pregnancy as a result of RCA, highlighting the compounded vulnerability to RCA and IPV occurring in the context of disability, and underscoring need for respectful and confidential RCA and IPV education and assessments during pregnancy as well as disability-specific resources and safety planning [96]. One quantitative study of college students identified having a health problem that required the use of “special equipment” as a risk factor for RCA (p = 0.049) [32]. A study of 5497 family planning clients revealed a significantly higher likelihood of disclosing RCA in participants with disabilities (p < 0.0001) [97]. One other qualitative study reported the number of women with disabilities in their sample (n = 4 of 14), but did not further explore this aspect of their RCA experience [26].

Heightened vulnerability to violence for women with disabilities has been explored more thoroughly in the literature [98, 99], and women with disabilities have been targeted historically by eugenicist reproductive control and forced sterilization efforts [100–102]. More work is needed to identify specific aspects of partner-initiated RCA that may differentially impact this population, or may require tailored educational resources, supports, and interventions.

### Can people assigned male at birth be RCA victims?

A common question in the field of RCA is whether people assigned male sex at birth (AMAB; cisgender men and transgender women) can be victims of RCA. Often cited is the idea of “entrapment”, in which people assigned female sex at birth (AFAB) trick a partner into getting them pregnant, usually to ensure connection or financial gains. This is highlighted in qualitative literature, as noted by this male participant in Alexander and colleagues’ 2019 study describing women who trick men into pregnancy: “﻿They got power over him because basically they’re saying, you got a baby, you gonna have to deal with her for the rest of his life because that’s your baby mama. You gone have to deal with her unless you don’t want to deal with the kid and then you gone have to deal with the law so basically, she got you trapped all the way around” [76]. The US population-based National Intimate Partner and Sexual Violence Survey (NISVS) measured RCA among both male and female respondents using two questions, asking did a partner ever “get you pregnant (if victim is female)/tried to get pregnant (if victim is male) when you did not want to become pregnant or tried to stop you from using birth control?” or ever “Refused to use a condom when you wanted them to use one?” [27]. Using this limited RCA construct, the survey revealed 8.4% of women reported lifetime RCA, and 9.7% of men [27]. This construct raises the question of whether condom refusal on the part of AFAB people is clearly a form of RCA (it is possible that the AFAB person is refusing a condom because they know they are using an alternative method of contraception such as pills or an implant). Additionally, given the risks associated with pregnancy and childbirth, and the disproportionate burden of parenting borne by AFAB people, one might ask whether the consequences of RCA for AMAB people are the same as the consequences for AFAB people. Coercion into pregnancy and parenting is certainly harmful to any person, but we argue that the experience of RCA for AMAB people is fundamentally different and not comparable to RCA experienced by AFAB people.

## Conclusion

The nascent field of RCA research has explored multiple new areas and provided insight into many aspects and levels of this form of coercion. In this evolving area of scholarship, there are numerous gaps and remaining questions to be answered as researchers seek to fine tune the concept and elucidate risks, protective factors, and areas for intervention. In the United States, eroding reproductive rights have shifted the landscape of pregnancy decision-making and brought autonomy and control over fertility to the forefront. Areas for future exploration include evolving interpretations of pregnancy intention in the setting of fewer options for abortion, RCA in people with disabilities and multiple levels of marginalization, including sexual and gender minorities; intersections between RCA and economic abuse in the context of efforts at economic justice; and community-centered approaches to intervention and prevention. Additionally, when considering solutions to RCA it is essential to reflect global contexts (such as preference for male children, involvement of family members, and preference for large families) and the need for tailored interventions. Reflecting the overall trend in IPV intervention and prevention, there is an effort to move away from a checklist-screening approach in which providers use validated questions and follow a protocol based on response, toward a model of trauma-sensitive care and offering strategies and resources to patients regardless of disclosure. Models of intervention strategies that include as well as move beyond harm reduction are needed to support those exposed to RCA as well to prevent such behaviors from occurring.

## Data Availability

Not applicable.
